# Motor Subtypes of Parkinson’s Disease Can Be Identified by Frequency Component of Postural Stability

**DOI:** 10.3390/s18041102

**Published:** 2018-04-05

**Authors:** Saba Rezvanian, Thurmon Lockhart, Christopher Frames, Rahul Soangra, Abraham Lieberman

**Affiliations:** 1 School of Biological and Health Systems Engineering, Arizona State University, Tempe, AZ 85281, USA; saba.rezvanian@asu.edu (S.R.); cframes@asu.edu (C.F.); 2 Barrow Neurological Institute, Phoenix, AZ 85013, USA; Abe.Lieberman@dignityhealth.org; 3 Department of Physical Therapy, Crean College of Health and Behavioral Sciences, Chapman University, Orange, CA 92866, USA; soangra@chapman.edu

**Keywords:** Parkinson’s disease (PD), tremor dominant (TD), postural instability and gait difficulty (PIGD), center of pressure (COP), fast Fourier transform (FFT), wavelet transform (WT)

## Abstract

Parkinson’s disease (PD) can be divided into two subtypes based on clinical features—namely tremor dominant (TD) and postural instability and gait difficulty (PIGD). This categorization is important at the early stage of PD, since identifying the subtypes can help to predict the clinical progression of the disease. Accordingly, correctly diagnosing subtypes is critical in initiating appropriate early interventions and tracking the progression of the disease. However, as the disease progresses, it becomes increasingly difficult to further distinguish those attributes that are relevant to the subtypes. In this study, we investigated whether a method using the standing center of pressure (COP) time series data can separate two subtypes of PD by looking at the frequency component of COP (i.e., COP position and speed). Thirty-six participants diagnosed with PD were evaluated, with their bare feet on the force platform, and were instructed to stand upright with their arms by their sides for 20 s (with their eyes open and closed), which is consistent with the traditional COP measures. Fast Fourier transform (FFT) and wavelet transform (WT) were performed to distinguish between the motor subtypes using the COP measures. The TD group exhibited larger amplitudes at the frequency range of 3–7 Hz when compared to the PIGD group. Both the FFT and WT methods were able to differentiate the subtypes. COP time series information can be used to differentiate between the two motor subtypes of PD, using the frequency component of postural stability.

## 1. Introduction

In 2010, approximately 630,000 people in the U.S. were diagnosed with Parkinson’s disease (PD)—a number that is estimated to double by 2040 [1]. PD is a progressive neurodegenerative disorder that includes motor and non-motor features [2]. PD can be further divided into two subtypes based on clinical features—namely tremor dominant (TD) and postural instability and gait difficulty (PIGD) [2,3,4,5]. This categorization is important at the early stage of PD, since identifying the PD subtypes can help to predict the clinical progression of the disease. Several studies have confirmed that the PIGD subtype has a faster disease progression and greater motor function impairment [6], and is less responsive to levodopa and deep brain stimulation when compared to the TD subtype [3,5,7,8]. It has also been reported that there is a correlation between the freezing of gait (FOG) score and the PIGD score [7]. Additionally, the PIGD subtype can place PD patients at a higher risk of falling when compared to TD patients [9]. It has been shown that PIGD patients have worse postural control when compared to TD patients [9,10]. Accordingly, correctly diagnosing subtypes can help caregivers to initiate early amenable interventions and track the progression of the disease. It should be noted that the diagnosis would not lead to a different medical treatment. However, another treatment needs to be taken alongside the medical treatment for PIGD patients in order to reduce the loss of balance and falling, since dopaminergic medications may result in limited improvement in postural instability and gait [11,12]. Thus, the diagnosis leads to the specific path that should be taken for the patient to manage the symptoms. 

The differentiation of TD from PIGD is currently based on sub-scores of the Unified Parkinson’s Disease Rating Scale (UPDRS) [3,11]. The UPDRS is scored by clinicians, and thus is subjective and prone to error [12]. Subtype-specific biomarkers may improve the accuracy of the diagnoses that are relevant to the PD subtypes and progression. 

The center of pressure (COP) measure is widely employed in assessing postural control, and has been utilized for analyzing the disease-related features in PD patients [13,14,15,16]. The results of different studies have indicated that COP was more variable for PD patients, relative to the control participants [14,15], and that COP-derived velocities were abnormally large for PD patients with FOG when compared to the patients without FOG [13]. Thus, COP is considered as a good measure for representing PD disease-related postural characteristics.

PD tremor is present while resting, and is typically dampened with kinetic movement. Therefore, in order to distinguish between the two subtypes, proposing a static test appears to be more appropriate than a dynamic task [17]. Several studies have reported a frequency range of 3–7 Hz for PD tremor [17,18,19]. It has also been demonstrated that the whole-body COP signal has a frequency lower than 2 Hz [20,21,22]. Subtype-specific postural instability in PD may be better identified by the frequencies that make up the COP signal. We hypothesized that the whole-body COP frequency may be a better and more objective means of identifying the PD subtypes. The most common method to investigate the tremor in PD is fast Fourier transformation (FFT) [5,6]. FFT transfers a signal from the time domain to the frequency domain. In this method, the time information is lost after the transformation. Therefore, a method such as wavelet transformation (WT)—which includes both the time and frequency information of the signal [7]—might help to diagnose the subtypes better than FFT. Based on the importance of correct PD subtype diagnosis and the lack of an objective method among the current diagnosis techniques, this study aims to develop an objective method to diagnose PD motor subtypes by employing COP data and using the FFT and WT methods.

## 2. Materials and Methods

### 2.1. Participants

Thirty-six participants that were diagnosed with PD by specialists at the Muhammad Ali Parkinson Center at the Barrow Neurological Institute (Phoenix, AZ, USA) were recruited for this study. The participants’ demographic information is presented in Table 1. The Movement Disorder Society Unified Parkinson’s Disease Rating Scale (MDS-UPDRS) was employed to identify the TD and PIGD groups [23]. The designated items for TD (kinetic and postural tremor in both the right and left hand; tremor—while at rest—of either the face and lips or the chain, arms, and legs) and PIGD (freezing, walking, posture, gait, and postural stability) were used to calculate the mean TD and PIGD scores. The ratio of the mean TD score to the mean PIGD score was used to identify the TD group. The patients with a ratio greater than or equal to 1.5 were classified as TD, while those with a ratio less than or equal to 1.0 were classified as PIGD. The patients with ratios ranging from 1.0 to 1.5 were classified as mixed-type, and were considered as an exclusionary criterion for this study [5,11,24].

The study was approved by the Institutional Review Board at the Barrow Neurological Institute and Arizona State University, Tempe, AZ, USA. The participants provided informed consent prior to their inclusion in the study. All of the assessments were performed while subjects were in the “on” medication status—approximately 1 to 1.5 h after taking the PD medication.

### 2.2. Experimental Procedure 

The participants were placed with their bare feet on the force platform and were instructed to stand upright with their feet shoulder width apart and their arms by their sides for 20 s, and look straight ahead during the experiment. They were instructed not to talk or bend their knees throughout the experimental trials. Harnesses were fitted onto the participants to avoid falls. This task was performed under two conditions—namely eyes open and eyes closed. For the eyes closed condition, the subjects were asked to close their eyes during the experiment. Each participant performed the experiment under both conditions. Each condition had three trials. 

### 2.3. Data Analysis 

COP data were derived using force plate data sampled at 100 Hz. Both anterior–posterior (AP) and medial–lateral (ML) COP data were low-pass-filtered using a fourth-order, zero lag Butterworth filter with a cut-off frequency of 10 Hz. Five traditional COP measures were calculated to assess whether or not the two subtypes of PD can be distinguished by using the time domain information. The measures included the following: COP range (the range of COP displacement), resultant COP path length (the total COP trajectory length), resultant mean velocity (the resultant path length divided by the total duration), and a 95% confidence ellipse area (the smallest ellipse that will cover 95% of the points of the COP diagram). Based on previous studies, these traditional parameters are good indicators of postural instability [14,25,26] and were considered as variables that might help us to distinguish PIGD from TD. All of the analyses were performed in MATLAB version 2015a.

### 2.4. TD vs. PIGD Detection Method

In order to distinguish between the TD and PIGD subtypes, the following two methods were utilized: fast Fourier transform (FFT) and wavelet transform (WT). In the FFT method, the PD subtypes were identified by the frequency spectra of COP signals. Two frequency bands were introduced [27,28,29]: the COP band and the tremor band. The COP and tremor bands were defined as the frequency components from 0–3 Hz to 3–7 Hz, respectively. The detection method was defined as the ratio of the area under the power spectra of the tremor band to the summation of the areas under the power spectra of the COP band and the tremor band. 

COP data were transformed into the wavelet domain using daubechies mother wavelet (db6). It was chosen because it has been widely employed in different human posture and movement studies [27,30]. Various mother wavelets were also applied to ensure that the optimal selection was made appropriately. The results supported the notion that daubechies mother wavelet was the best choice. In the WT method, the COP and tremor bands were defined as the scales that corresponded to the frequency ranges of 0–3 Hz and 3–7 Hz, respectively. The detection method was defined in a similar manner to the way it was defined in the FFT method: the ratio of the averaged WT coefficients of the tremor band to the summation of the averaged WT coefficients of the COP band and the tremor band. This ratio was unitless because it was a ratio of values with the same unit. In both methods, the defined ratio was multiplied by 100 in order to obtain a value between 0 and 100. Values that were closer to 100 indicated a higher possibility of the TD subtype, while the possibility of the PIGD subtype increased as the values approached 0. The first time derivative of COP time series was defined as COP velocity (V-COP). The ratio that was defined above was applied to COP (R_COP_) and COP velocity (R_VCOP_) in both the AP and ML directions. 

### 2.5. Statistical Analysis

Analysis of variance (ANOVA) with repeated measures on the traditional COP measures and the proposed detection ratio (using both the FFT and WT methods) were performed. Different factors—such as condition (two levels: eyes open (EO) and eyes closed (EC)) and group (or subtype) of PD (two levels: TD and PIGD)—were considered as within-subject and between-subject factors, respectively. Comparisons of interest exhibiting statistically significant differences (*p* < 0.05) were further analyzed using post hoc tests with Bonferroni corrections. In all analyses, sphericity assumptions were tested (Greenhouse–Geisser analysis). The diagnostic performance of the proposed method—or the accuracy of a test to discriminate between the subtypes—was further evaluated using receiver operating characteristic (ROC) curve analysis [31] for the directions and factors of both methods. In a ROC curve, the true positive rate (sensitivity) is plotted as a function of the false positive rate (100—specificity) at different cut-off points. Therefore, each point on the ROC curve corresponds to a sensitivity/specificity pair for a particular decision threshold. Therefore, the upper-left corner denotes a test with perfect discrimination (no overlap in the two distributions) in a ROC curve analysis. Accordingly, the closer the ROC curve is to the upper-left corner, the higher the overall accuracy of the test [31]. In this study, PD subtypes were diagnosed by utilizing UPDRS and were considered as a correct diagnosis. All of the statistical analyses were performed based on this assumption. In all tests, *p* < 0.05 was considered as a significant level. Statistical analyses were performed using IBM SPSS Statistics 22.

## 3. Results

The results of the traditional COP measures—under both the eyes open and eyes closed conditions—are provided in Table 2. All of the variables had larger values in the eyes closed condition compared to the eyes open condition. Because these parameters did not have a normal distribution, a Box-Cox transformation was applied and parametric methods were performed. There was no significant difference between the two groups for all the variables. However, there was a significant difference between the conditions for all of the parameters (range AP: F_(1,34)_ = 4.252, *p* = 0.047; range ML: F_(1,34)_ = 60.34, *p* = 0.001; path length: F_(1,34)_ = 29.797, *p* = 0.001; mean velocity: F_(1,34)_ = 29.795, *p* = 0.001; area: F_(1,34)_ = 11.847, *p* = 0.002). 

**Figure 1:**
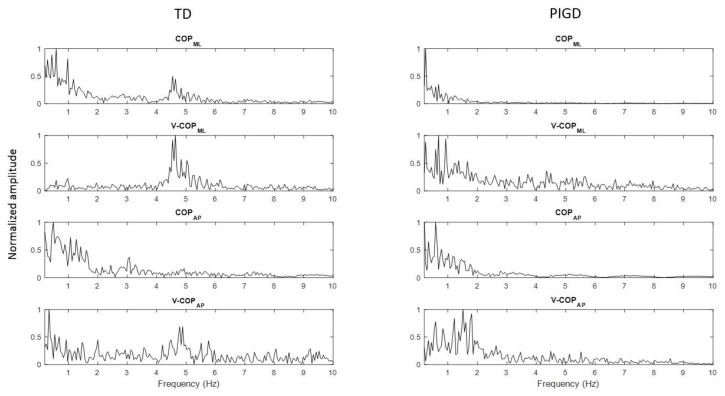
Power spectrum of COP and COP velocity of a tremor dominant (TD) patient and a postural instability and gait difficulty (PIGD) patient for both the medial–lateral (ML) and anterior–posterior (AP) directions. The graphs on the left and right sides of the page present the power spectrum signal of a TD patient and a PIGD patient, respectively. COP_ML_: COP in the ML direction, COP_AP_: COP in the AP direction, V-COP_ML_: COP velocity in the ML direction, and V-COP_AP_: COP velocity in the AP direction.

A power spectral analysis of the COP and COP velocity of a TD patient and a PIGD patient are plotted in Figure 1, revealing that both patients had frequency components ranging from 0 to 2 Hz in their COP and COP velocity signals. However, only the TD patient had an increase in power spectrum in the frequency band of 3–7 Hz. This increase was larger in the ML direction.

**Figure 2:**
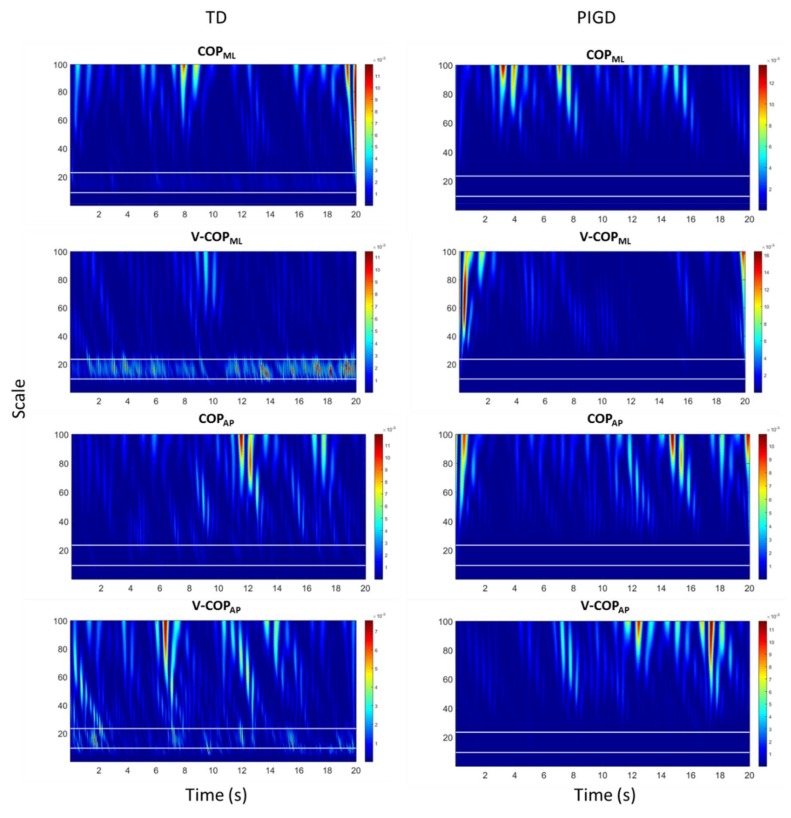
Wavelet transform (WT) of COP and COP velocity of a TD patient and a PIGD patient for both the ML and AP directions. The horizontal white lines in each plot indicate the PD tremor scale range corresponding to the frequency range of 3–7 Hz. The frequencies of 3 Hz and 7 Hz correspond to the scales of 24 and 10, respectively. COP_ML_: COP in the ML direction, COP_AP_: COP in the AP direction, V-COP_ML_: COP velocity in the ML direction, and V-COP_AP_: COP velocity in the AP direction.

The WT of COP and COP velocity of a TD patient and a PIGD patient in both the ML and AP directions are plotted in Figure 2. The horizontal white lines in each figure indicate the PD tremor scale range corresponding to the frequency range of 3–7 Hz. The WT coefficients in Figure 2 display relatively larger values in the PD tremor scale range (i.e., lighter blue values appeared in between two horizontal white lines) for the TD patient when compared to the PIGD patient. Similar to the power spectral analysis (Figure 1), these increases were larger in the ML direction.

**Figure 3:**
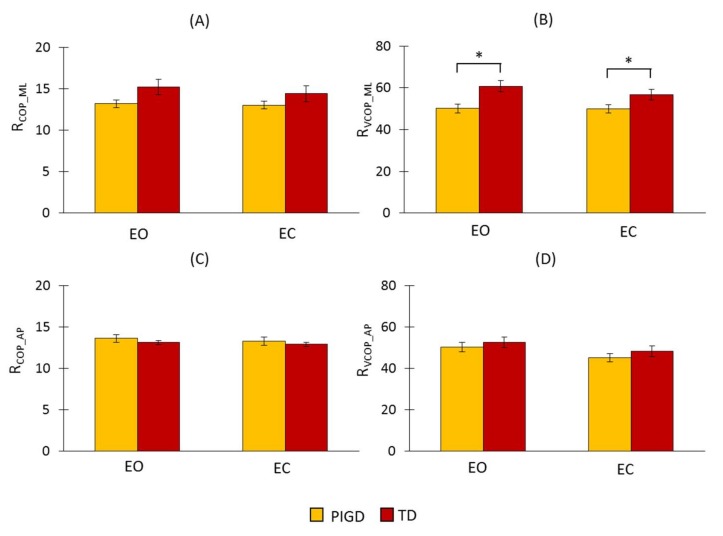
Fast Fourier transform (FFT) results of the proposed detection ratio for COP and its velocity in both the ML and AP directions. (**A**) R_COP_ML_: the detection ratio using COP data in the ML direction, (**B**) R_VCOP_ML_: the detection ratio using COP velocity data in the ML direction, (**C**) R_COP_AP_: the detection ratio using COP data in the AP direction, and (**D**) R_VCOP_AP_: the detection ratio using COP velocity data in the AP direction. The asterisks (*) placed over the vertical bars denote a significant difference (*p* < 0.05). EC: eyes closed condition; EO: eyes open condition.

The results of the proposed detection ratio for COP and its velocity in both directions using FFT are presented in Figure 3. Neither the ratio of COP (R_COP_ML_) nor its velocity (R_VCOP_ML_) in the ML direction were significantly different across the different conditions (R_COP_ML_: F_(1,34)_ = 2.006, *p* = 0.112; R_VCOP_ML_: F_(1,34)_ = 2.67, *p* = 0.112). However, a statistically significant difference in R_VCOP_ML_ across the groups (F_(1,34)_ = 7.978, *p* = 0.008) was observed, although no significant difference was found in R_COP_ML_ (F_(1,34)_ = 3.449, *p* = 0.072). In both R_COP_ML_ and R_VCOP_ML_, there was no significant interaction between the condition and the group (R_COP_ML_: F_(1,34)_ = 1.181, *p* = 0.285; R_VCOP_ML_: F_(1,34)_ = 2.037, *p* = 0.163). R_VCOP_ML_ was larger for the TD group than for the PIGD group (Figure 3A,B). This indicated that there were larger amplitudes in the frequency range of 3–7 Hz in this group. In the AP direction, there was no significant difference across the groups (R_COP_AP_: F_(1,34)_ = 0.498, *p* = 0.485; R_VCOP_AP_: F_(1,34)_ = 0.628, *p* = 0.433) and the conditions (R_COP_AP_: F_(1,34)_ = 1.306, *p* = 0.201; R_VCOP_AP_: F_(1,34)_ = 3.45, *p* = 0.08) in both R_COP_AP_ and R_VCOP_AP_.

**Figure 4:**
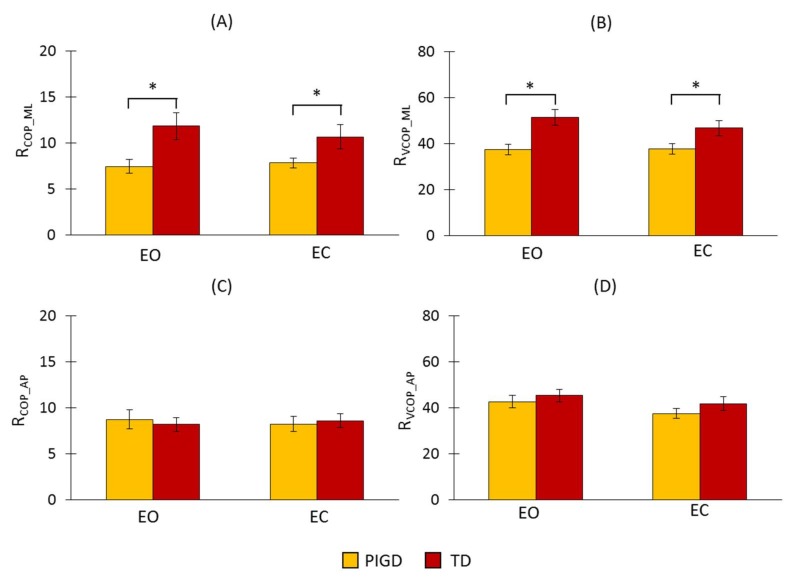
WT results of the proposed detection ratio for COP and its velocity in both the ML and AP directions. (**A**) R_COP_ML_: the detection ratio using COP data in the ML direction, (**B**) R_VCOP_ML_: the detection ratio using COP velocity data in the ML direction, (**C**) R_COP_AP_: the detection ratio using COP data in the AP direction, and (**D**) R_VCOP_AP_: the detection ratio using COP velocity data in the AP direction. The asterisks (*) placed over the vertical bars denote a significant difference (*p* < 0.05).

The explained WT method was applied to COP and its velocity in both directions. The results are presented in Figure 4. We found a significant difference between the groups for R_COP_ML_ and R_VCOP_ML_ (R_COP_ML_: F_(1,34)_ = 7.589, *p* = 0.009; R_VCOP_ML_: F_(1,34)_ = 10.066, *p* = 0.003), but no significant difference between the conditions (R_COP_ML_: F_(1,34)_ = 0.373, *p* = 0.814; R_VCOP_ML_: F_(1,34)_ = 2.5, *p* = 0.123). There was no significant interaction between the conditions and the groups (R_COP_ML_: F_(1,34)_ = 3.044, *p* = 0.09; R_VCOP_ML_: F_(1,34)_ = 2.828, *p* = 0.102). Both R_COP_ML_ and R_VCOP_ML_ had larger values for the TD group than for the PIGD group (Figure 3A,B). These increases occurred because of the larger amplitude values in the scales corresponding to the frequency range of 3–7 Hz. In the AP direction, there were no significant differences across the groups (R_COP_AP_: F_(1,34)_ = 0.004, *p* = 0.952; R_VCOP_AP_: F_(1,34)_ = 0.854, *p* = 0.362) or conditions (R_COP_AP_: F_(1,34)_ = 0.011, *p* = 0.916; R_VCOP_AP_: F_(1,34)_ = 3.047, *p* = 0.091) in both R_COP_AP_ and R_VCOP_AP_.

**Figure 5:**
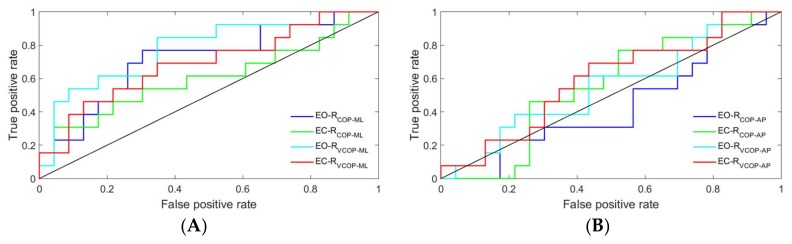
Receiver operating characteristic (ROC) curves of the proposed detection ratio using the FFT method for COP and its velocity: (**A**) ML direction and (**B**) AP direction. EO-R_COP_ML_: the detection ratio using COP data in the ML direction under the eyes open condition, EC-R_COP_ML_: the detection ratio using COP data in the ML direction under the eyes closed condition, EO-R_VCOP_ML_: the detection ratio using COP velocity data in the ML direction under the eyes open condition, EC-R_VCOP_ML_: the detection ratio using COP velocity data in the ML direction under the eyes closed condition, EO-R_COP_AP_: the detection ratio using COP data in the AP direction under the eyes open condition, EC-R_COP_AP_: the detection ratio using COP data in the AP direction under the eyes closed condition, EO-R_VCOP_AP_: the detection ratio using COP velocity data in the AP direction under the eyes open condition, and EC-R_VCOP_AP_: the detection ratio using COP velocity data in the AP direction under the eyes closed condition.

**Figure 6:**
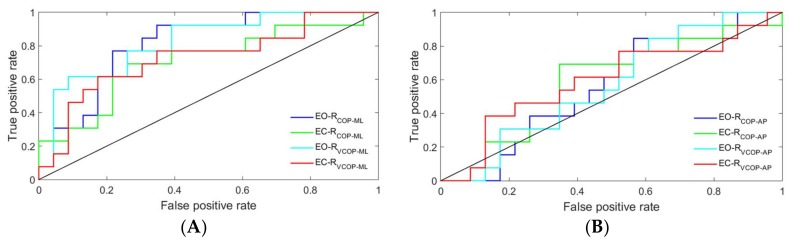
ROC curves of the proposed detection ratio using the WT method for COP and its velocity: (**A**) ML direction and (**B**) AP direction. EO-R_COP_ML_: the detection ratio using COP data in the ML direction under the eyes open condition, EC-R_COP_ML_: the detection ratio using COP data in the ML direction under the eyes closed condition, EO-R_VCOP_ML_: the detection ratio using COP velocity data in the ML direction under the eyes open condition, EC-R_VCOP_ML_: the detection ratio using COP velocity data in the ML direction under the eyes closed condition, EO-R_COP_AP_: the detection ratio using COP data in the AP direction under the eyes open condition, EC-R_COP_AP_: the detection ratio using COP data in the AP direction under the eyes closed condition, EO-R_VCOP_AP_: the detection ratio using COP velocity data in the AP direction under the eyes open condition, and EC-R_VCOP_AP_: the detection ratio using COP velocity data in the AP direction under the eyes closed condition.

The ROC curves of the proposed detection ratio for COP and its velocity in both directions and under both conditions are plotted in Figure 5 and Figure 6 for the FFT and WT methods, respectively. In both methods, the ROC curves were closer to the upper-left corner in the ML direction than they were in the AP direction, which indicated a higher overall accuracy of the test in the ML direction [31].

The ROC curves were further analyzed by calculating the areas under each curve. The results are presented in Table 3. Only COP velocity data in the ML direction could significantly distinguish between the two subtypes using the FFT method. The results of the area under the ROC curves also revealed that the WT method could significantly distinguish between the two subtypes by using either COP or COP velocity data in the ML direction, regardless of the conditions. 

## 4. Discussion

This study addressed subtype-specific biomarkers in order to classify the inherent heterogeneity of PD. This categorization can help to predict the clinical progression of the disease. Thus, the correct diagnosis of the subtypes can assist caregivers in initiating early amenable interventions and managing symptoms. The COP time series of PD patients were analyzed to distinguish between the two subtypes of PD. To the best of our knowledge, this study is the first to attempt to objectively diagnose the TD and PIGD subtypes of PD. Postural stability is maintained through neuromuscular feedback loops and open loop control processes that constantly adapt to internal and external perturbations [32,33]. Utilizing specific statistical and numerical tools, these control mechanisms can be quantified to identify neuromuscular changes that occur with pathology. Thus, traditional linear postural measures and Fourier transformation were applied to the COP time series and the increment of the COP time series in both the AP and ML directions. Furthermore, in order to quantify the changes in COP dynamics that occur at multiple timescales, a wavelet transform was employed to infer the underlying nature and control mechanisms involved in balance maintenance and the disease state. 

In the traditional measures of postural sway, the parameters that denoted the magnitude of the postural movements were unable to discriminate between the TD and PIGD subtypes (Table 2). However, when visual information was occluded, a coincident decrease in postural stability was reflected in both subtypes for the linear postural measures (i.e., COP range, mean velocity, path length, and a 95% confidence ellipse area). These results were consistent with previous investigations regarding postural stability in PD patients [34]. 

Both the power spectral density and the WT of the COP time series and its velocity (Figure 1 and Figure 2) revealed an increase in the 3–7 Hz frequency range of the TD group, a frequency spectra that is reportedly symptomatic of parkinsonian tremor [17,18,19]. In fact, the ML COP data exhibited a greater frequency content than the AP COP data, which was consistent with previous investigations, which reported that PD patients exhibited increased ML sway amplitude, decreased AP sway amplitude, and possibly postural inflexibility in the AP direction [15,35,36,37]. In this context, the preponderance of the ML frequency in the ML direction, coupled with the impaired movement in the AP direction, suggested an underlying postural inflexibility in PD patients, where the tremor reflected in the ML time domain might be a consequence of the AP direction’s inability to contain movements in a higher frequency range [35,38,39]. Our proposed ratio was not able to show a statistically significant difference between the TD and PIGD patients in the AP direction using either of the methods—even accounting for both COP and COP velocity. The reason was that the tremor frequency had a larger amplitude in the ML direction than it did in the AP direction (as shown in Figure 1 and Figure 2). However, both the FFT and WT methods were able to distinguish the TD patients from the PIGD patients using the ML-COP velocity signal, while only the WT method was able to specify the subtype with the COP position time series. This could be explained by the fact that the FFT method used only the frequency information from the signals, while the WT method employed both the frequency and time components. The information from the signals that was utilized by WT enabled us to specify the subtypes of PD using both COP and COP velocity. Additionally, FFT displayed significant results when it employed COP velocity—as opposed to COP in itself—because the velocity of the signal was a first time derivative of the signal, which captured more variation of the signal. Hence, FFT could assess more information about the signals when it employed COP velocity. The results of the proposed method were consistent across both conditions (EO and EC) in both methods (FFT and WT). This consistency indicated the strength of the proposed diagnostic method using the proposed ratio. Although the proposed method can distinguish the TD from the PIGD subtypes, further studies are required to define the threshold value ranges that can classify the patients.

## Figures and Tables

**Table 1:** Demographics of the tremor dominant (TD) and postural instability and gait difficulty (PIGD) groups (mean ± standard deviation—SD). MDS-UPDRS: Movement Disorder Society Unified Parkinson’s Disease Rating Scale.

	TD (*n* = 13)	PIGD (*n* = 23)
Gender (F:M)	0:13	9:14
Age (years)	59.92 ± 9.63 (34–71)	70.43 ± 6.18 (59–81)
Disease duration (months)	20.23 ± 19.14 (4–60)	37.78 ± 54.69 (1–216)
MDS-UPDRS III (ON)	14.85 ± 9.85	15.08 ± 8.48

**Table 2:** Selected postural stability parameters. Range anterior–posterior (AP): center of pressure (COP) range in the AP direction, range medial–lateral (ML): COP range in the ML direction, path length: resultant COP path length, mean velocity: resultant COP mean velocity, and area: 95% ellipse area. The symbols * or ** denote which of the two variables were significantly different at each parameter (*p* < 0.05).

		Range AP (cm)	Range ML (cm)	Mean Velocity (cm/s)	Path Length (cm)	Area (cm^2^)
Eyes open	TD	0.81 ± 0.15 *	1.49 ± 0.10 *	1.46 ± 0.27 *	29.28 ± 5.49 *	0.92 ± 0.22 *
	PIGD	1.06 ± 0.13 **	1.81 ± 0.16 **	1.48 ± 0.23 **	29.62 ± 4.57 **	1.53 ± 0.32 **
Eyes closed	TD	1.15 ± 0.24 *	2.81 ± 0.39 *	2.56 ± 0.66 *	51.23 ± 13.16 *	2.95 ± 1.05 *
	PIGD	1.14 ± 0.14 **	2.75 ± 0.36 **	2.01 ± 0.20 **	40.23 ± 4.01 **	2.51 ± 0.45 **

**Table 3:** The area under the receiver operating characteristic (ROC) curves of the proposed detection ratio, using both the FFT and WT methods, for COP and its velocity in the ML and AP directions under the two conditions (eyes open (EO) and eyes closed (EC)). The *p*-values of each parameter are presented in parentheses. The asterisks (*) indicate that the area under the ROC curve was significantly different from 0.5 (*p* < 0.05).

		FFT		WT	
		COP	V_COP	COP	V_COP
ML-Direction	EO	0.689 (*p* = 0.05)	0.779 * (*p* = 0.001)	0.809 * (*p* = 0.001)	0.823 * (*p* = 0.001)
	EC	0.602 (*p* = 0.343)	0.712 * (*p* = 0.023)	0.706 * (*p* = 0.033)	0.726 * (*p* = 0.016)
AP-Direction	EO	0.562 (*p* = 0.542)	0.555 (*p* = 0.5873)	0.562 (*p* = 0.529)	0.569 (*p* = 0.482)
	EC	0.555 (*p* = 0.578)	0.592 (*p* = 0.358)	0.579 (0.442)	0.595 (*p* = 0.363)
